# Surveillance of Child and Youth Mental Disorders and Associated Service Use in Canada

**DOI:** 10.1177/07067437231182059

**Published:** 2023-06-26

**Authors:** Jordan Edwards, Paul Kurdyak, Charlotte Waddell, Scott B. Patten, Graham J. Reid, Leslie Anne Campbell, Katholiki Georgiades

**Affiliations:** 1 Department of Psychiatry and Behavioural Neurosciences, 3710 McMaster University, Hamilton, Ontario, Canada; 2 Offord Centre for Child Studies, 3710 McMaster University, Hamilton, Ontario, Canada; 3 Department of Psychiatry, 7938 University of Toronto, Toronto, Ontario, Canada; 4 Institute of Mental Health Policy, Management, and Evaluation, University of Toronto, Toronto, Canada; 5 Children's Health Policy Centre, Faculty of Health Sciences, 1763 Simon Fraser University, Vancouver, British Columbia, Canada; 6 Cuthbertson & Fischer Chair in Pediatric Mental Health, Department of Community Health Sciences, 2129 University of Calgary, Calgary, Alberta, Canada; 7 Departments of Psychology & Family Medicine, 6221 The University of Western Ontario, London, Ontario, Canada; 8 459884 Children's Health Research Institute, London, Ontario, Canada; 9 Department of Community Health and Epidemiology, 3688 Dalhousie University, Halifax, Nova Scotia, Canada

**Keywords:** surveillance, child and adolescent psychiatry, mental health services, epidemiology, community mental health services, administrative data, population-level surveys, data linkage, methods

## Introduction

Public health surveillance is defined as the continuous and dynamic process of observing and measuring a target in a specific population over time and space. It includes continuous measurement, data collection, analysis, interpretation, knowledge production, and dissemination to a directed audience including policymakers.^1^ The ultimate goal of public health surveillance is to generate timely evidence to inform public health responses designed to improve population health.^2^

Mental disorders, in aggregate, are the leading contributors to disability in children and youth worldwide.^3^ Unlike other noncommunicable diseases, which start later in life, approximately half of all mental disorders emerge prior to age 18.^4^ These disorders are commonly untreated and often persist into adulthood contributing to significant impairment over the life course.^3^

In Canada, over the past decade, mental ill-health among youth and young adults aged 12 to 24 has increased, particularly symptoms of depression, anxiety, suicidality, and substance use, disproportionately impacting females.^5^ In Ontario, perceptions of the need for professional help for mental health concerns among children and youth aged 4 to 16 years tripled from 6.8% to 18.9% between 1983 and 2014.^6^ Evidence also suggests large and disproportionate mental health treatment gaps, impacting children and youth from immigrant families, and females with mood and/or anxiety disorders.^7^

Despite these concerning trends, Canada has yet to establish a comprehensive, national approach to the surveillance of mental disorders and associated service use among children and youth.

Current evidence is fragmented and disparate, reflecting heterogeneity in (a) data sources, including both general, population-based surveys and health administrative data; (b) geographical coverage, comprising evidence from local, regional, provincial/territorial, and national levels; and (c) conceptualization and measurement, including measures of perceptions of treatment need, symptoms, functioning, and diagnoses. Furthermore, current surveillance methodology has been largely limited to the use of singular data types (i.e., survey or health administrative data) in isolation (see Supplement 1). We believe there is a need for a national surveillance strategy for children and youth that concerns the unique aspects of this population. Compared to the general population, children and youth require distinct measurement approaches which vary across developmental stages (i.e., preschool-age, children, and youth). Measurement must take into consideration the onset of specific classes of disorders across developmental stages, which require information from multiple informants (youth, parent, and teacher) to be accurate.^8^ Furthermore, it is required to capture relevant sectors providing mental health care for children and youth, inclusive of school-based services.

**Figure 1.**
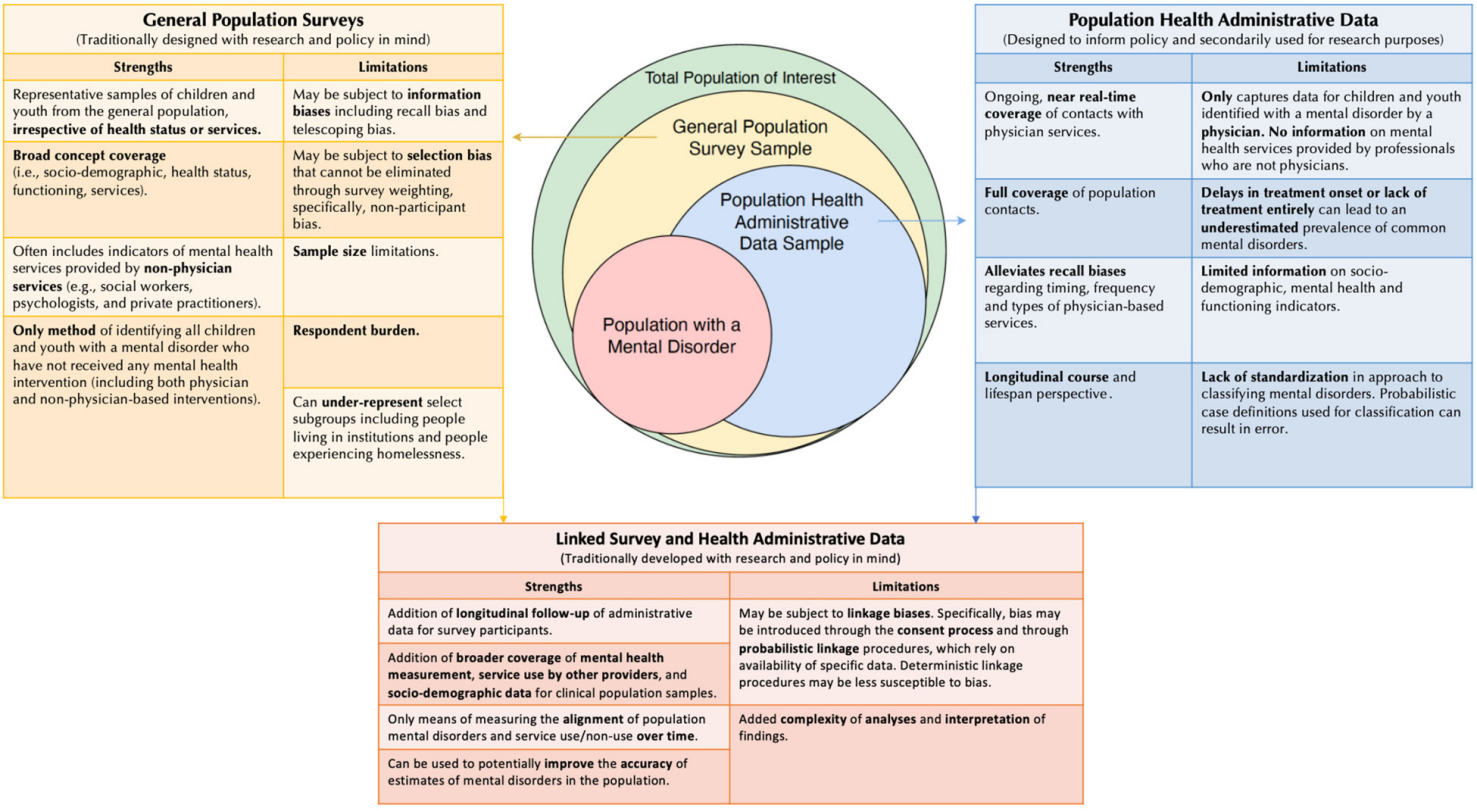
Using population surveys and health administrative data to monitor child and youth mental disorders and associated service use. The three boxes present the strengths and limitations of (a) general population surveys; (b) health administrative data; and (c) the linkage between these two data sources to monitor child and youth mental disorders and associated service use. The Venn diagram in the centre describes a theoretical estimate of different proportions of the population of children and youth (depicted as overlapping circles). The outer circle of the diagram (green) describes the entire population of interest, and the inner circles reflect samples of children and youth ascertained through the various data collection methodologies. The yellow circle represents general population survey samples, and the blue circle represents population health administrative data samples. The red overlapping circle represents the sample of the total population of interest with a mental disorder that is captured to varying degrees by general population survey sampling and population health administrative data.

Both survey and health administrative data provide distinct coverage of the population and are characterized by a diverse range of strengths and limitations for measuring mental disorders at the population level (see Figure 1). One key distinction between these data sources is that both capture different estimates of prevalence. Specifically, survey data is used to estimate the population prevalence of mental disorders (i.e., an estimate that can be generalized back to the target population). In contrast, health administrative data is most appropriately used to estimate the contact or treated prevalence of mental disorders (i.e., an estimate of who contacts, or who is treated in the health care system (often limited to physicians) with signs or symptoms of a mental disorder).^9^

Rather than relying on *either* general population-based surveys *or* health administrative data for surveillance purposes, recent methodological and data analytic advances have highlighted the benefits of linking survey and health administrative data to generate prevalence estimates of mental disorders and service utilization.^10^ Linked data can be used to overcome certain limitations present in each data type, including adding longitudinal follow-up of administrative data for survey participants and adding more comprehensive coverage, such as standardized measures of mental disorders, service use by other providers, and sociodemographic data for health administrative data. Data linkages offer the ability to improve both the temporal coverage and the accuracy of various indicators that have been proposed to be essential components for informing mental health care systems.^10,11^ For example, the accuracy of mental health-related service use among children and youth, commonly measured using health administrative data alone, would be improved if estimates of nonphysician and private practitioner service use, obtained through survey data, or from institutions themselves, were integrated using data linkages. Furthermore, administrative-survey data linkage is currently our best means of measuring the alignment of population mental disorders and service use/nonuse over time, which is an important indicator measuring potential gaps between need and use that could be used to inform policymakers.^7,11,12^ It is important to note that the introduction of data linkage brings with it unique challenges and potential sources of bias arising both from the survey and administrative data. Specifically, recall bias can affect the validity of survey-based measures and probabilistic classification of administrative data (e.g., using probabilistic case definitions) can result in error.^13^ Moreover, misclassification of mental health-related service contacts for children and youth can arise when parents or caregivers seek care for a child with mental health concerns on their own. In addition, bias can arise from the linkage process itself, which is determined by a combination of participant consent and patterns of missing data, both of which can potentially impact the representativeness of linked cohorts (see Figure 1).^14^ Here, we outline current surveillance efforts, highlight content and methodological gaps, and propose a vision for the future of surveillance of child and youth mental disorders and associated service use in Canada.

## Current Surveillance Methodology

### Data Sources

In Canada, as described above, data used to inform surveillance efforts fall into two categories: general population-based survey data (hereafter called *survey data*) and *health administrative data*. The collection of national or provincial *survey data* is most often led by Statistics Canada (StatCan), a government-funded organization with legislated, federal responsibility to collect data and generate population estimates. StatCan is responsible for Canada's census and conducts regular surveys on a range of topics, including health, to support decision-makers across the country. StatCan collaborates with other governmental and pan-Canadian organizations, as well as academic researchers to develop the content of their health surveys.^15^ *Health administrative databases*, in comparison, are held and managed nationally at the Canadian Institute for Health Information (CIHI), a federal organization.^15^ A subset of data that is collected, housed, and used at the provincial/territorial level for planning purposes are sent to CIHI. These include physicians’ billing data and associated physician and patient demographic data. Note that there exist fragmented physician-based records sent to CIHI from Quebec. While hospitalization records are not sent from Quebec to CIHI, since 2018/2019, emergency department data has been sent to CIHI from the Ministère de la Santé et des Services Sociaux du Québec. CIHI is responsible for coordinating and developing a national integrated approach to health information with the goal of providing timely and relevant data to inform health policy and healthcare delivery.^15^

In recent years, collaborations between StatCan and CIHI have led to linkages between surveys and various health administrative databases. Though these linkages provide unique advantages over individual data sources,^12^ they are not yet used in Canadian surveillance.

### Surveillance Methodology

Current mental disorder-related surveillance efforts have primarily been developed around indicators derived from singular data types (i.e., survey or health administrative data). The standard approaches are to either use multiple iterations of large cross-sectional surveys, or multiple years of administrative data. Approaches have focused on estimating prevalence and incidence, stratified by various sociodemographic factors. Regional variation is often limited to the provincial/territorial level.^16^

#### Examples of Mental Disorder Surveillance Approaches in Canada Relevant to Children and Youth

The Public Health Agency of Canada has led surveillance efforts in the form of: (a) the Canadian Chronic Disease Surveillance System (CCDSS) established in 2009,^16^ which synthesizes provincial/territorial health administrative data providing annual estimates of service use for mental health-related concerns among those ages 1 and older across Canada, all-cause mortality following first health care encounter for certain mental disorders, and estimates of the incidence, prevalence, and all-cause mortality among people ages 10 and older meeting the case definition for having schizophrenia obtained through physician contacts; and (b) the Positive Mental Health Surveillance Indicator Framework (PMHSIF) established in 2016,^17^ which synthesizes multiple sources of survey data (e.g., Canadian Community Health Survey and the Canadian Health Survey on Children and Youth), providing evidence on levels of positive mental health across all ages and associated risk and protective factors in the population.

CIHI has led surveillance activities, including the development of periodic reports (latest in 2022) on health topics, including child mental disorders.^18^ Similar to estimates provided from the CCDSS, a number of CIHI reports focus on mental health-related service contacts among those ages 5 to 24 years; these have focused on contact with physicians in various settings including hospitals—and for certain provinces/territories, emergency departments, and outpatient settings. CIHI also synthesizes data on some services not provided by physicians, including from the Kids Help Phone and from medication dispensation records from some, but not all, provinces/territories.^18^

Other national surveillance initiatives have been developed by the Canadian Primary Care Sentinel Surveillance Network (CPCSSN), which was established in 2008 and uses electronic medical records from participating primary care clinicians across Canada to inform disease surveillance across all age groups. The primary indicator, used by the CPCSSN, for mental health-related surveillance is depression, which has an available case-finding algorithm based on physician diagnoses and medications prescribed.^19^ Though we have focused our writing on national efforts, we recognize there exist numerous provincial/territorial-level surveillance efforts that offer important regional insights (see Supplement 1).

Surveillance activities have also been conducted by academic researchers across Canada using different data sources and methodologies over the past two decades. Examples include (a) an approach developed by Goldner et al^9^ in 2003 using health administrative data from British Columbia to estimate the prevalence of schizophrenia-related disorders among those between the ages of 15 and 65 years; (b) an approach led by Kisely et al^20^ in 2009 using administrative data from five provinces across Canada for surveillance of mental disorders across all ages; (c) an approach developed by Lesage et al^21^ in 2015 for Quebec, which builds on data used in the CCDSS to examine mortality rates among people ages 1 and older with a mental disorder (ascertained through physician-based contacts) in Quebec using health administrative data linked with the vital statistics death database; (d) an approach led by Anderson et al^22^ in 2019, which used community-based early psychosis client and physician-level data linked with health administrative data to measure regional incidence of first-episode psychosis among those between the ages 16 and 50 years; and (e) an approach led by Vigo et al^23^ in 2021, which proposes to triangulate estimates of mental and substance use disorders among those ages 15 and older using a combination of Canadian health administrative and survey data, in addition to international evidence. To date, most academic pursuits have been limited to individual provinces/territories due to the challenges of coordinating and sharing data across provinces/territories. Work from the Health Data Research Network Canada^24^ is hoping to reduce these limitations by making cross-provincial/territorial data sharing easier, which may offer insight for future mental health-related surveillance efforts.

It is important to note that while several surveillance efforts include children and youth, the PMHSIF, CPCSSN, and periodic reports from CIHI are the only national surveillance initiatives that explicitly include a focus on children and youth. Given that PMHSIF is limited to positive mental health indicators ascertained through survey data and that evidence from CIHI and CPCSSN is limited to physician-based health data, it is evident that Canadian efforts have yet to leverage the strengths of linked survey and administrative data sources to establish a more rigorous approach to surveillance of child and youth mental disorders.

## Evidence Gaps

There is currently a lack of consensus on how to measure and quantify mental disorders at the population level. As a result, we lack robust empirical evidence to answer fundamental questions related to the *number of children and youth in Canada with a mental disorder*. Furthermore, we have no centralized way of tracking the *numbers seen by service providers for mental health-related concerns*, inclusive of all sectors that serve this population, that is, health, education, community mental health and social services, and justice services.^7^ Another important gap is our capacity to conceptualize and measure the *reach and targeting of mental health services, including the alignment of “need” for mental health services and service receipt—*defined as the proportion of children with and without a mental disorder who have and have not received mental healthcare.^11,12^ These 3 core concepts—prevalence of mental disorders, service use, and reach and alignment of mental health services—represent critical information gaps that are central for informing policymaking.^11^ These gaps exist for both national and provincial/territorial approaches to surveillance.

## Vision for the Future of Surveillance

Current surveillance in Canada lacks the specificity required to meaningfully inform child and youth mental health policy. We must both create high-quality population-level data and make better use of the data we currently have, to support policymakers as they plan to address the population-based needs for mental health services for children and youth in Canada. The development of analytic methods that combine multiple sources of data, including individually linked high-quality survey and health administrative data, is also needed to arrive at valid and generalizable estimates.

Future surveillance initiatives for child and youth mental disorders should be supported by federal leadership and facilitated by collaborative cross-sectoral and interprovincial/territorial partnerships. We need to include a diverse range of young people and caregivers with lived experiences, clinicians, senior administrators, researchers, and policymakers to help ensure the data are appropriate and useful for all stakeholders. The national leadership of surveillance efforts has numerous strategic advantages including collaborative partnerships across the country and the establishment of national standards for conceptualizing, measuring, collecting, appraising, integrating, and interpreting child and youth mental health data. Examples of successful national surveillance efforts include major disease surveillance systems for cancer and diabetes (see Supplement 1).

### Development of a Coordinated and Collaborative Surveillance Platform in Canada

Child and youth mental disorder surveillance in Canada would benefit from the development of a coordinated and collaborative platform dedicated to child and youth mental disorders that include the following priorities.

- Establish best practices for evaluating, appraising, and using Canadian data to inform current surveillance efforts and future mental health data collection. There is a need to identify current evidence gaps and reduce them by evaluating and leveraging the strengths of the diverse range of data sources and data linkages currently available for estimating mental disorders in Canada. In support of this goal, Canada should look to bring key stakeholders together, from across the country, to come to consensus on a standardized set of indicators for population measurement of mental disorders and associated mental health service use among children and youth. Furthermore, Canada can learn and integrate methodologies from successful global surveillance efforts including work conducted in the global burden of disease study^25^ and evidence from individual countries. For example, the UK has prioritized the collection of high-quality prevalence data of mental disorders among children and youth, disaggregated by key sociodemographic factors, every three years beginning in 2021, acknowledging the importance of routinely collected and timely data for informing policy.^26^ Canada faces significant temporal gaps in available mental health data for children and youth. As such, we can learn from these international efforts and establish an approach for collecting and using population-level data to inform policy and reduce mental health disparities on an ongoing basis.
- Compile and characterize current approaches to surveillance and data sources in an open and living space. Building on prior provincial/territorial efforts to characterize data sources,^27^ Canada could develop a national-level repository that is updated when new data become available. Currently, barriers exist for accessing, linking, and vetting data, which may be contributing to an underutilization of existing population-level data in Canada. The reduction in barriers to accessing population-level data across Canada requires the collaboration of various federal and provincial/territorial organizations and ministries of health to come together. One promising development is the recent priority setting by Health Canada in February 2023, which outlines a goal of working towards shared and standardized health indicators, including those for mental health.^28^ The report also eludes to the creation of a Centre of Excellence of health workforce data with the goal of building capacity for the use of data to improve health care.^28^ We hope this work incorporates increasing access to data to support surveillance efforts.
- Developing approaches to surveillance that best serves the information needs of mental health policymakers and people with lived experiences. By working closely with provincial/territorial mental health policymakers and people with lived experiences, to understand their collectively informed needs, Canada can ensure that mental health data will be used and will be useful for evidence-informed mental health policies. As policy needs may vary regionally over time, surveillance may be most informative if it is supported by underlying theoretical and analytical infrastructure that is flexible and adaptable over time to be contextually relevant and useful.
- Using the surveillance platform to advance research. The development of a surveillance platform can also support the advancement of innovative surveillance-related methodology, including approaches to conceptualize, measure, model, and communicate findings on mental disorders at the population level. Further, these approaches can inform our understanding of mental disorders in the population by identifying risk and protective factors, in addition to evaluating policy impact at the population level.

## Conclusion

Given increases in mental ill-health,^5^ associated impairments,^3^ and service shortages^29^ affecting Canadian children and youth—and when considering current and future economic costs^30^ of mental disorders—it is imperative that we invest now in evidence-informed mental health policy by building capacity for the collection, use, and integration of mental health data for children and youth in Canada. Surveillance is at the heart of understanding health conditions and is key to informing policymakers as they address population-based needs. It is time to prioritize child and youth mental health in Canada by investing in developing, sustaining, and continuously enhancing a robust mental disorder surveillance system developed explicitly for children and youth.

## Supplemental Material

sj-docx-1-cpa-10.1177_07067437231182059 - Supplemental material for Surveillance of Child and Youth Mental Disorders and Associated Service Use in Canada Click here for additional data file. Supplemental material, sj-docx-1-cpa-10.1177_07067437231182059 for Surveillance of Child and Youth Mental Disorders and Associated Service Use in Canada by Jordan Edwards, Paul Kurdyak, Charlotte Waddell, Scott B. Patten, Graham J. Reid, Leslie Anne Campbell and Katholiki Georgiades in The Canadian Journal of Psychiatry
