# Supplementary material for: Surveillance of Child and Youth Mental Disorders and Associated Service Use in Canada
Source: Can J Psychiatry. 2023 Jun 26;68(11):819–25. doi: 10.1177/07067437231182059 (PMC10590091; doi:10.1177/07067437231182059)
Supplement: sj-docx-1-cpa-10.1177_07067437231182059 - Supplemental material for Surveillance of Child and Youth Mental Disorders and Associated Service Use in Canada [file sj-docx-1-cpa-10.1177_07067437231182059.docx]

Supplement 1: Additional Resources.

**National/inter-provincial Evidence:**

- Canadian Institute for Health Information. Children and Youth Mental Health. Available from: https://www.cihi.ca/en/children-and-youth-mental-health-in-canada
- Canada PHA of. Report from the Canadian Chronic Disease Surveillance System: mental illness in Canada 2015. 2015;
- Health Data Research Network [Internet]. Available from: https://www.hdrn.ca
- Kisely S, Lin E, Lesage A, Gilbert C, Smith M, Campbell LA, et al. Use of administrative data for the surveillance of mental disorders in 5 provinces. Can J Psychiatry. 2009; Sunderland A, Findlay LC. Perceived need for mental health care in Canada: results from the 2012 Canadian community health survey-mental health. Statistics Canada Ottawa; 2013.
- Lix LM, Ayles J, Bartholomew S, Cooke CA, Ellison J, Emond V, et al. The Canadian chronic disease surveillance system: a model for collaborative surveillance. Int J Popul Data Sci. 2018;3(3).
- Orpana H, Vachon J, Dykxhoorn J, McRae L, Jayaraman G. Monitoring positive mental health and its determinants in Canada: the development of the Positive Mental Health Surveillance Indicator Framework. Heal Promot chronic Dis Prev Canada Res policy Pract. 2016;36(1):1.
- Queenan JA, Wong ST, Barber D, Morkem R, Salman A. The Prevalence of Common Chronic Conditions Seen in Canadian Primary Care. 2021;

***British Columbia Evidence:***

- Goldner EM, Jones W, Waraich P. Using administrative data to analyze the prevalence and distribution of schizophrenic disorders. Psychiatr Serv. 2003;54(7):1017–21.
- Vigo D, Jones W, Dove N, Maidana DE, Tallon C, Small W, et al. Estimating the prevalence of mental and substance use disorders: a systematic approach to triangulating available data to inform health systems planning. Can J Psychiatry. 2022;67(2):107–16.
- Waddell C, Catherine N, Krebs E, Nosyk B, Cullen A, Hjertaas K, et al. Public Data Sources for Monitoring Children ’ s Mental Health : What We Have and What We Still Need in British Columbia. 2020;(December):1–23.

***Alberta Evidence:***

- Alberta Interactive Health Data Application. Available from http://www.ahw.gov.ab.ca/IHDA_Retrieval/

***Manitoba Evidence:***

- Manitoba Centre for Health Policy [Internet]. Available from: https://umanitoba.ca/faculties/health_sciences/medicine/units/chs/departmental_units/mchp/index.html

***Ontario Evidence:***

- Anderson KK, Norman R, Macdougall AG, Edwards J, Palaniyappan L, Lau C, et al. Estimating the incidence of first-episode psychosis using population-based health administrative data to inform early psychosis intervention services. 2019;40.
- Boyle MH, Georgiades K, Duncan L, Comeau J, Wang L. The 2014 Ontario Child Health Study—Methodology. Can J Psychiatry [Internet]. 2019;64(4):237–45. Available from: https://doi.org/10.1177/0706743719833675.
- Yang J, Kurdyak P, Guttmann A. Developing Indicators for the Child and Youth Mental Health System in Ontario. Healthc Q. 2016.

***Quebec Evidence:***

- Institute National de Santé Puplique du Québec [Internet]. Available from: https://www.inspq.qc.ca
- Lesage A, Rochette L, Émond V, Pelletier É, St-Laurent D, Diallo FB, et al. A surveillance system to monitor excess mortality of people with mental illness in Canada. Can J Psychiatry. 2015;60(12):571–9.

***Successful Surveilance Efforts in Cancer and Diabetes:***

- Brenner DR, Poirier A, Woods RR, Ellison LF, Billette J-M, Demers AA, et al. Projected estimates of cancer in Canada in 2022. CMAJ. 2022;194(17):E601–7.
- Johnson KC. National Enhanced Cancer Surveillance System: a federal-provincial collaboration to examine environmental cancer risks [Status report]. Chronic Dis Inj Can. 2000;21(1):34.
- LeBlanc AG, Gao YJ, McRae L, Pelletier C. At-a-glance-Twenty years of diabetes surveillance using the Canadian Chronic Disease Surveillance System. Heal Promot Chronic Dis Prev Canada Res Policy Pract. 2019;39(11):306.
